# Autologous Materials in Regenerative Dentistry: Harvested Bone, Platelet Concentrates and Dentin Derivates

**DOI:** 10.3390/molecules25225330

**Published:** 2020-11-15

**Authors:** Sara Bernardi, Guido Macchiarelli, Serena Bianchi

**Affiliations:** 1 Department of Life, Health and Environmental Sciences, University of L’Aquila, 67100 L’Aquila, Italy; gmacchiarelli@univaq.it (G.M.); serena.bianchi@univaq.it (S.B.); 2 Centre of Microscopy, University of L’Aquila, 67100 L’Aquila, Italy

**Keywords:** autologous materials, regenerative medicine, bone regeneration

## Abstract

The jawbone is a peculiar type of bone tissue, unique for its histological, anatomical and physiological characteristics. Therefore, a defect in the maxilla or in the mandible, because of pathological sequelae is difficult to prevent and to restore. Several biomaterials have been and are currently being developed to respond to the demands of regenerative medicine. A specific group of biomaterials used in regenerative dentistry is represented by the autologous materials. Platelet concentrates harvested bone and dentin derivates are indeed used in an attempt to minimise the alveolar resorption or in vertical ridge augmentation procedures or in sinus lift interventions. The aim of this review is to examine the properties of the above-listed materials, to compare them and to indicate eventual clinical applications.

## 1. Introduction

The bone of maxillary and mandible arches possesses a unique and fascinating biomorphology which differs from the bone physiology of the skeletal system [1]. These differences are due to the different functional roles that the oral cavity, whose jaws represent the skeletal support, play during the human lifetime [2]. The most evident functionality is the masticatory one, due to the articulation of the alveolar bone with the teeth, but also the phonatory, respiratory, sensory and physiognomic roles are important [3].

Infective, inflammatory, and traumatic pathologies can affect the teeth, and as a consequence, also the support alveolar bone, which tends to resorption, especially when the dental element is lost [3]. Reasons for the tooth loss are to be searched in dental tissues’ pathologies, however, those that produce important inflammatory sequelae such as periodontitis and traumas significantly affect the degree of bone loss around the tooth [4].

Even though the condition of tooth-lessness, known as edentulism, is not life-threatening, but it affects the quality of life of patients [5]. Patients nowadays demand a fixed long-lasting prosthodontic solution, but the placing of implants requires adequate anatomical conditions which are not always available. Indeed traditional implantology relies on placing a fixture longer than 10 mm with a diameter measuring more than 3.5 mm [6], and both jaws, maxillary and mandible, host important anatomical structures, such as the maxillary sinus, with its rich vascular supply [7], and the inferior alveolar nerve in case of the mandible bone [8]. If the bone volume is not adequate to host the fixture placement, the above-mentioned structures can be damaged with permanent and serious consequences on the quality of life of the patients.

The restoration of an adequate bone volume to properly host implants capable of mechanically supporting fixed prostheses requires the development of surgical techniques and performing grafts [9]. The surgical regeneration techniques vary according to the clinical situation: in maxillary arch, e.g., the sinus lift with lateral and crestal approach is used to gain a vertical ridge augmentation [10]. When the width of the arch is not adequate, split-crest techniques can be used [11]. In the mandible, the vertical ridge augmentation is quite challenging, even though the “sandwich” technique has been reported as one of the safest and most reliable [12].

Due to the morbidity risks related to the surgical interventions, a parallel arm of research on short implants is currently on-going [13,14,15]. In their recent clinical report, Naenni et al. highlighted how the use of implants of 6 mm length did not show significant differences in survival rate and peri-implant marginal bone loss compared to implants of 10 mm length [15]. The Group 1 ITI Consensus Report in 2018 [16] also stated that the use of shorter implants was a valid alternative to the placement of standard-length implants when bone volume is not adequate. The statement regarding the survival rate on short implants has the support of meta-analysis based on randomised clinical trials (RCTs).

Surgical regeneration success depends on three main factors, i.e., the experience and the skills of the surgeon, the biological response of the patient and the quality of the grafting materials. The ideal qualities required in the best grafting material for bone regenerative medicine are osteoconduction, osteogenesis and osteoinduction [17].

**Figure 1:**
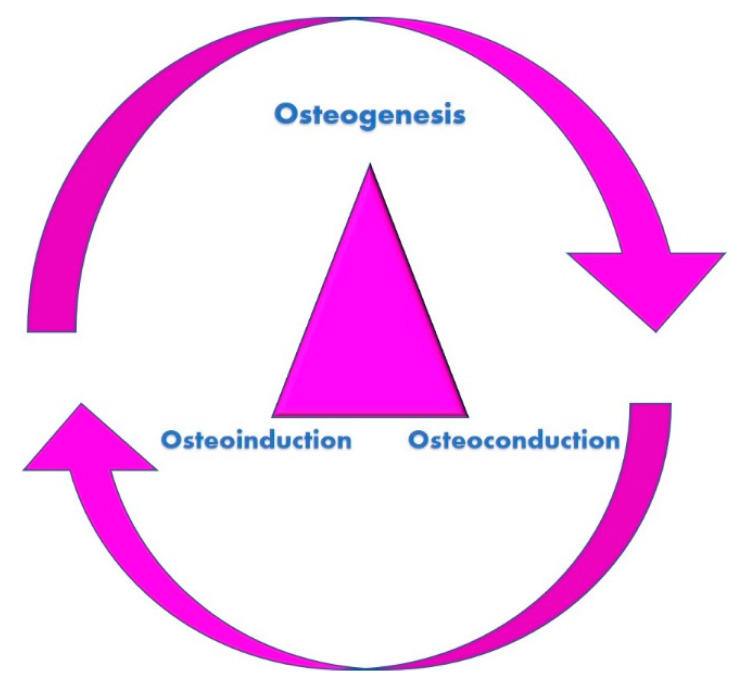
The three properties fundamental for bone regeneration. The scaffold network allows the migration of the cells (osteoconduction). Subsequently, the growth factors provided by the vascular supply induce the cellular proliferation (osteoinduction). Finally, the osteoblasts form new bone tissue (osteogenesis).

The osteoconduction properties are provided by the scaffolds, which represent the structures that allow cellular replication and tissue development. The osteogenic properties are due to the presence of osteoblasts producing the extracellular matrices and the osteoinduction is stimulated by the growth factors starting a series of events leading to the regeneration process [14] (Figure 1).

So far, the material that has all these properties is autologous bone [18]. The availability of autologous bone is not taken for granted and substitutes have been developed and tested with some success degrees. In the field of autologous grafts, not only the bone tissue has been explored but also tissues with similar components (dentin and enamel derivates) [19] and rich in growth factors (platelets concentrates) [20] have been used and currently tested for regenerative purposes.

The aim of this review is to examine the autologous bone harvested from other sites, the platelet concentrates and the dentin derivates, to compare their properties and to evaluate their possible clinical indications.

## 2. Autologous Bone

**Figure 2:**
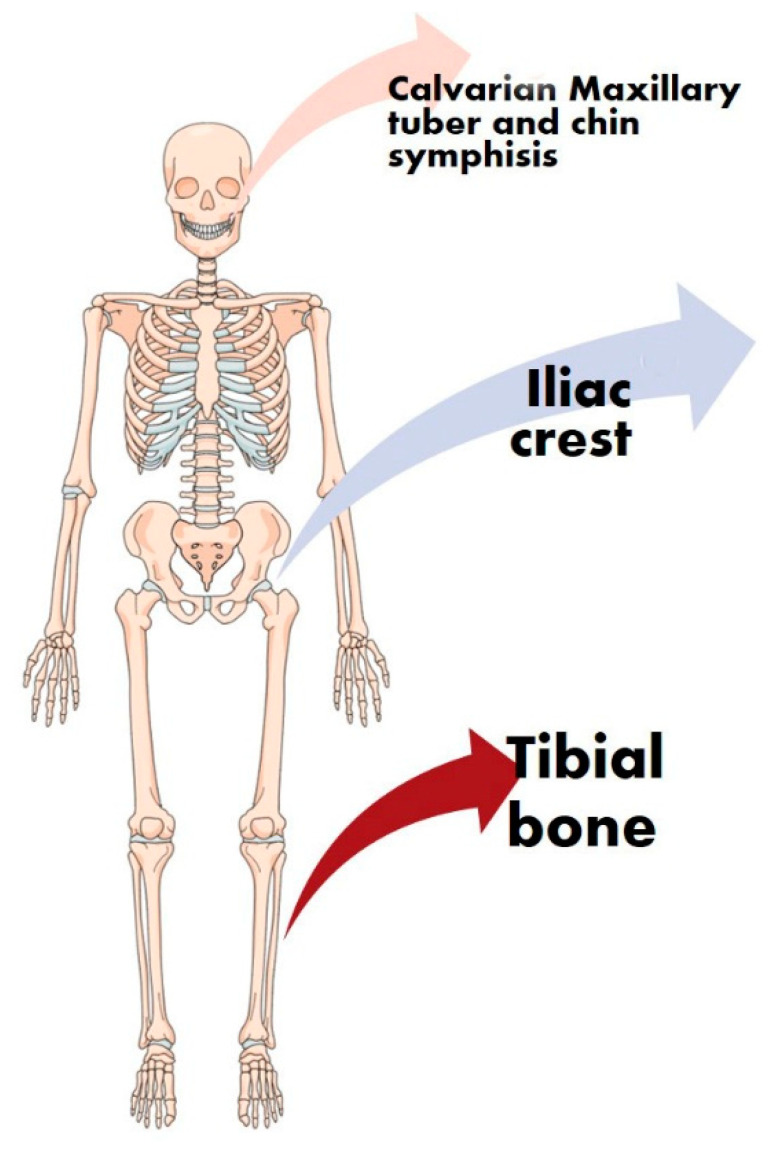
Schematic representation of the harvesting sites of autologous bone. All of the bones used as a donor site belong to skeletal areas important for the quality of life of the patients. The orange arrow indicates those sites from the head skeleton (calvarian, maxillary tuberosity and chin), the blue arrow indicates those sites from the pelvis (iliac crests) and the red arrows indicate those sites from the leg (tibial bone).

Autologous bone refers to bone tissue harvested from a site of the same patient. Autologous bone for orthopaedic and reconstruction of the facial bones is taken from the iliac crest, from tibial bone, the mandibular ramus, the chin symphysis and maxillary tuber (Figure 2) [21].

Autologous bone owns all three properties required for regenerations: indeed, the structure of the bone serves as a physical support for the osteoconduction, as a proper scaffold. The morphology of the cortical graft is indeed optimal to mechanically support that will be colonised by the cellular elements [18]. It is obvious to state that the autologous harvested bone contains a certain quantity of vital osteoblasts. The cells represent the portion of the material for the osteogenesis, necessary for the cellular proliferation and the production of new bone tissue in the recipient sites [22]. Finally, the vascular supply of the recipient sites and the harvested biomaterial will provide the growth factors needed for the osteoinduction [23].

Possessing all these properties and belonging to the same patient, autologous bone is considered the gold standard material in bone regeneration procedures. Indeed, the risks of transmitted diseases and immunological reactions are unlikely [18]; however, the risk of morbidities related to the harvesting interventions and the related costs make its use for great and important reconstructions (such as mandible restoring after head and neck cancer) [22].

Indeed, the interventions of bone harvesting are associated to hematoma, nervous injures, fractures of the bone used as donor site (pelvis and mandible) [24], infections, limitations of the functionality and chronic pain.

## 3. Autologous Platelet Concentrates

Clinicians rely on the use of autologous platelet concentrates or as a form of natural “scaffold,” thanks to the network of fibrin fibres, or in combination with other biomaterials, due to their evident osteoconductive and osteoinductive properties [25].

In the last few years, different kinds of protocols have been developed to obtain autologous platelet concentrates: the first one was platelet-rich plasma (PRP) and plasma rich in growth factors (PRGF) [26]. This first attempt to obtain a material rich in growth factors, however, presented several challenging factors in its preparation, limiting its use. Indeed, the protocol for the PRP production included the blood withdrawn by venipuncture in tubes with acid citrate dextrose, a first centrifugation, the transfer of the obtained supernatant into other tubes, a second centrifugation at higher speed, the removal of the obtained supernatant and the gentle resuspension of the plasma clot [27]. The preparation, therefore, was complicated and hardly reproducible.

The factors limiting the use and the versatility of PRP and PRGF induced the necessity of a second APCs generation: platelet-rich-fibrin (PRF) [28]. The protocol for obtaining PRF is relatively simpler than PRP, with no need for blood biochemical manipulation, resulting in an easy-to-use product. The preparation of PRF includes the solely use of centrifuge (the original protocol proposed by Choukroun et al. included a speed centrifugation of 3000 rpm for 10 min), as a result, giving a different quality of the polymerised fibrin [28] compared to PRP.

One of the derived PRF products is the concentrated growth factors (CGF), which, as a result of their high-density tetramolecular matrix fibrin, contain high levels of growth factors [29]. The protocol of centrifugation used to obtain CGF starts with 30 s of acceleration, 2 min at 2700 rpm, 4 min at 2400 rpm, 4 min at 2700 rpm, 3 min at 3000 rpm to finish with 36 s of deceleration [29]. The different speeds of centrifugation determines the production of a high-density matrix, rich in growth factors, as previously reported in immunohistochemical studies [29]. The advantages of using CGF in regenerative procedures is the slow release of growth factors improving the healing process [30].

**Figure 3:**
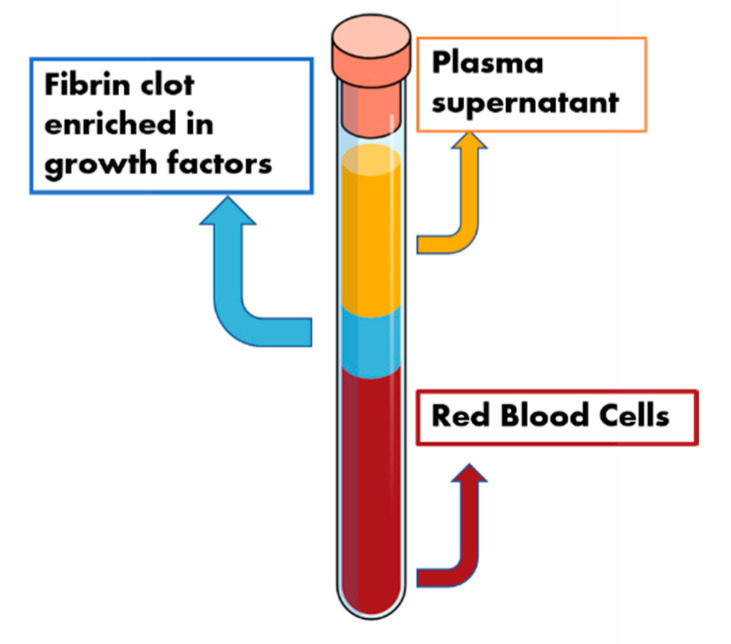
Schematic representation of the autologous platelets concentrates. Red blood cell bodies can be found on the bottom of the centrifugation product. The middle portion is composed of the “scaffold” portion (fibrin fibres) and the induction molecules (growth factors). The upper portion is represented by the supernatant plasma proteins.

Autologous platelet concentrates are exploited as a biomaterial in regenerative dentistry due to the presence of fibrin fibres and the high content of growth factors (Figure 3). The obtained fibrin is represented by a three-dimensional matrix, where platelets, glycanic chains and cytokines structural glicoprotein are entrapped [31]. The fibrin fibres, therefore, constitute a network suitable for cellular growth and represent the osteoconductive scaffold.

The growth factors, activated by the polymerisation of the blood clot and their own regeneration properties increase the reparative mechanism in the wound healing processes. The growth factors released by the autologous platelets concentrates are platelet-derived growth factor (PDGF), transforming growth factor b (TGF-b), insulin-like growth factor (IGF), epidermal growth factor (EGF), fibroblast growth factor (FGF) and bone morphogenetic protein (BMP) [23].

PDGF stimulates cell proliferation and collagen synthesis in fibroblasts; TGF-β induces the expression of extracellular matrix proteins, affects osteoblasts in an early stage of development and simulates collagen synthesis by fibroblasts; IGF helps differentiation and stimulates osteoblasts proliferation and differentiated functions such as type I collagen expression [23].

BMPs are a family of signalling molecules stimulating the formation of new bone also in heterotopic sites. The autologous platelet concentrates, in particular, contain BMP-2, which is capable of inducing the osteoblast differentiation [32,33].

The listed growth factors represent the osteoinductive elements.

## 4. Autologous Dentine Derivates

Another autologous material tested for alveolar bone regeneration is dentin and all of its derivates [19]. Indeed, dentin is a tissue much more available for dentists than the blood or the bone harvested from other sites, and the chemical composition and the embryologic origin make it a sort of “cousin” of the bone tissue [34].

The dentin is composed of a mineral phase (70%), organic matrix (20%) and water (10%) and by the tubule of odontoblasts that embryologically derive from a mesenchymal tissue [35]. The mineral phase is based on Ca-P (calcium-phosphate) molecules in four different forms (amorphous calcium phosphate, hydroxyapatite, tricalcium phosphate and octacalcium phosphate), whereas the organic matrix is composed by collagen I fibres and proteins such as growth factors, including bone morphogenetic proteins [35]. Therefore, it appeared that this kind of tissue could be a valid grafting material. In addition, since the dentin is a mineralised tissue, three forms of grafting material can be obtained and used for regeneration purposes, i.e., the mineralised matrix of dentine, the partially demineralised dentin matrix and the demineralised matrix of dentine [36].

The mineralised matrix of dentin provides a stable scaffold with good osteoconductive properties, but low osteoinductive potential. The demineralisation of the dentinal matrix, using a solution of 2% HNO_3_ [36], instead makes the material suitable for osteoinduction purposes, with a low osteoconduction property. Like the platelet concentrates, since the dentin misses the cellular bodies, the osteogenetic property is missing also in this autologous biomaterial.

## 5. Comparisons and Clinical Considerations

In situations requiring a regenerative technique, a clinician can choose among several biomaterials according to the clinical conditions, i.e., the requirements of the patient, in order to try to find a balance between the costs and the benefits.

The autologous class of biomaterials have the benefit to belong to the same patient, avoiding any possibility of immune reaction [37]. Using autologous bone is still the gold standard for the regeneration process that may still be an odd choice. The surgical harvesting procedures doubles the risks of postsurgical comorbidities. These considerations make the decision of choosing this type of biomaterial just for the reconstruction of extended areas such as the horizontal or the vertical ramus of the mandible or the extended volume of maxillary, and not for small edentulism [18].

Therefore, the choice largely relies on the platelet concentrates or the dentin derivates. As summarised in Table 1, the former would be particularly indicated for periodontal surgery, stimulating the fibroblasts present in the receiving site and using the fibrin network as a scaffold [20]. Among the generations of autologous platelet concentrates, if the aim is to rapidly stimulate the proliferation of the cells, the PRF would be more indicated. Instead, if the aim is to provide a more solid scaffold, represented by the fibrin network, and a retarded cell proliferation, CGF would be a better choice. PRP would be more indicated if the scaffold is supposed to remain for a longer period and if a faster regeneration is not required [30]. However, the difficulties in the preparation protocol of the PRP, make this choice unlikely.

Numerous studies reported the clinical use of autologous platelet concentrates mainly in grafting procedure and reconstruction of periodontal defects. Castro et al. performed a systematic review and meta-analysis on the use of PRF in periodontal defects [38]. They found 14 clinical studies whose outcomes showed how PRF had positive effects on the healing process of both hard and soft tissues [38]. Liu et al., in their recent meta-analysis, reported five randomised clinical trials using the PRF in maxillary sinus augmentation; the conclusion of the meta-analysis was that PRF might aid in reducing the healing time, but did not significantly improve the sinus lift [39]. Lately, Canellas et al., in their meta-analysis, considered the use of PRF in different oral procedures such as third molar extraction, sinus augmentation and the treatment of the implant marginal bone resorption. The meta-analysis process included 13 studies, and the reported results suggested the positive use of PRF in alveolar socket preservation, in reducing the marginal bone resorption around implants and to speed the sinus lift healing [40].

Alternatively, and if available, the use of autologous dentin derivates can be used, especially for the socket preservation. If a more stable scaffold is required, to facilitate the placement of a fixture after the extraction, e.g., the mineralised dentine would be a better choice [36]. If instead, the defect to be regenerated is periodontal, or to gain alveolar ridge in width and height, the demineralised dentine [41] would be the ideal choice. Indeed, demineralised dentine matrix, preserving the collagen fibres, containing high levels of growth factors, biglycans and collagen fibres promotes the formation of new bone tissue [42]. In particular, the biglycans, as reported by Avery et al., play a key role in the proliferation of osteoblast [42].

The main procedures reported by the few randomised controlled trials in literature are resumed in Table 2, using dentine derivates as socket preservation, guided bone regeneration and sinus augmentation [41,43,44,45,46,47]

## 6. Conclusions

The research towards biomaterials capable of replacing autologous bone and with osteogenetic properties still has not found a reliable alternative. However, in cases of regenerative dentistry, where the defects are limited in the extension, autologous platelets and autologous dentine derivates represent interesting choices to reach the regeneration purposes.

## Figures and Tables

**Table 1:** Table resuming the autologous biomaterial, their properties regarding the bone regeneration, the problem related to their use, and eventual clinical indication.

	Features	Osteo-Induction	Osteo-Conduction	Osteo-Genesis	Eventual Related Issue to Usage	Clinical Indications
Material						
**Autologous bone**		**+**	**+**	**+**	Comorbidities related to the surgical harvesting intervention, scarceness	Extended reconstruction of the jaws after head and neck cancers, important traumas
**Autologous platelet concentrates**		**+**	**+**	**-**	Blood manipulation, laws regarding the blood manipulation in private practices, obtaining protocol, skills in clot manipulation	Periodontal regeneration of alveolar defects. In combination with osteoconductive grafts, offer a more solid scaffold to the cellular migration and proliferation
**Autologous dentine derivates**		**+**	**+**	**-**	Availability of an extracted tooth Different protocols of obtainments Quantity of the graft that can be obtained from a tooth	Mineralised dentin matrix can be used in cases where a solid osteoconductive scaffold is required (e.g., for an early implant placement) Demineralised dentin matrix can be used to stimulate the osteoinduction but with low osteoconductive expectation (e.g., for the alveolar socket preservation)

**+** symbol indicates the owned properties. **-** symbol indicates the property lacks.

**Table 2:** Table resuming the clinical trials available in the literature on the use of dentine derivates.

Study	Type of Used Dentin	Interventions
Kim et al. (2010) [43]	Demineralised dentin	Guided bone regeneration
Jeong et al. (2011) [44]	Demineralised dentin	Sinus lift
Kim et al. (2016) [45]	Demineralised dentin	Guided bone regeneration
Pang et al. (2017) [46]	Demineralised dentin	Guided bone regeneration
Li, et al. (2018) [47]	Demineralised dentin	Guided bone regeneration
Minetti et al. (2020) [41]	Demineralised dentin vs. mineralised dentin	Alveolar preservation
